# Gut microbiota composition differences are associated with geographic location and age in malaria-endemic regions of Rwanda

**DOI:** 10.1371/journal.pone.0320698

**Published:** 2025-06-03

**Authors:** Jean d’Amour Mutoni, Matthias Van Hul, Aline Uwimana, Camille Petitfils, Giselle C. Wong, Anthony Puel, Amandine Everard, Hélène Alexiou, Leon Mutesa, Jean-Paul Coutelier, Nadine Rujeni, Patrice D. Cani

**Affiliations:** 1 Louvain Drug Research Institute (LDRI), Metabolism and Nutrition Research Group (MNUT), UCLouvain, Université catholique de Louvain, Brussels, Belgium; 2 Biomedical Laboratory Sciences Department, College of Medicine and Health Sciences, University of Rwanda, Kigali, Rwanda; 3 WELBIO Department, Walloon Excellence in Life Sciences and BIOtechnology (WELBIO), WEL Research Institute, Wavre, Belgium; 4 Dietetics Department, Haute Ecole Leonard de Vinci, Health Sector, Brussels, Belgium; 5 Centre for Human Genetics, College of Medicine and Health Sciences, University of Rwanda, Kigali, Rwanda; 6 De Duve Institute, UCLouvain, Université Catholique de Louvain, Brussels, Belgium; 7 Institute of Experimental and Clinical Research (IREC), UCLouvain, Université Catholique de Louvain, Brussels, Belgium; University of Uyo, NIGERIA

## Abstract

Evidence suggests that a significant interplay exists between the host gut microbiota and both the transmission and severity of malaria. Therefore, we explored the association between malaria and the gut microbiota across various geographic regions, considering host’s nutritional habits, helminth coinfections and age. This observational study was conducted in 3 malaria-endemic provinces of Rwanda: West, South and East. Demographic data, blood and fecal samples were collected from 169 participants (85 females and 84 males) aged between 2–78 years. We used questionnaire-derived qualitative data based on geographic regions, age, and nutrition. Malaria and soil-transmitted helminth diagnosis was assessed by microscopy. The gut microbial composition was analyzed based on bacterial 16S rRNA gene amplicon sequencing. We observed that preschool children had a significantly lower microbiota diversity compared to both school children (q = 0.027, K-Wallis) and adults (q = 0.011, K-Wallis). Unlike age, infection status (uninfected, malaria alone, soil-transmitted helminth alone or coinfection) was not significantly associated with the gut microbiota. However, using Bray-Curtis distances, we found a significantly differential gut microbial beta-diversity with a convergent distribution in the Western province compared to the other provinces (q = 0.0045, pairwise PERMANOVA). This geographic difference was not explained by any change in energy intake, protein, lipids, or carbohydrates consumption but was likely due to lower dietary fibre intake in the West compared to the South (q < 0.0001, ANOVA) and the East (q = 0.07, ANOVA). In conclusion, we have not found significant links between infection and gut microbiota. However, we showed a significant difference in the gut microbiota composition of people living in different geographic locations in Rwanda, possibly due to their nutritional habits.

## Introduction

Malaria is classified among the so-called group of ‘poverty-related diseases’, representing a major health problem predominantly in the global south [1]. The World Health Organization (WHO) reported 249 million cases and 608,000 deaths due to malaria worldwide in 2022 [2]. *Plasmodium falciparum* is the primary cause of severe malaria and is responsible for more than 90% of global malaria fatalities, with the Sub-Saharan African (SSA) region carrying over 90% of the burden [2,3].

The gut microbiota has been shown to play a major role in health and disease [4]. In the context of malaria, existing evidence shows trends but no causal roles have been established. A groundbreaking study published in 2014 discussed the role of gut microbiota-elicited alpha-gal antibodies in blocking *Plasmodium* transmission [5]. The latest publications of 2023 presented the genus *Bacteroides* as a key player in predisposing hosts to severe malaria in both human and murine subjects [6,7]. Importantly, the malaria-gut microbiota associations may be shaped by several factors such as geographic location, nutrition, coinfections (e.g., soil-transmitted helminths), age, antimalarials, deworming and antibiotic exposure [7–14].

Geographic variation is a critical factor shaping the host microbiota diversity and composition [15,16]. Specifically, in malaria-gut microbiota research, Yooseph et al. have shown differential microbiome composition by geographic regions ranging from Mali to Malawi and around the world [8]. According to the Center for Disease Control and Prevention (CDC, Atlanta, Georgia, USA), malaria distribution has wide geographic variations, even within a country [17]. In Rwanda, malaria predominantly occurs in the West, South and the East provinces [18], but there are no studies analyzing gut microbial composition in these regions.

Malaria-microbiome interactions may be influenced by coinfection with Soil Transmitted Helminths (STH) resulting in divergent and still poorly understood effects on gut bacteria [9,10]. Indeed, STH are among the most common parasitic infections worldwide and their distribution overlap with that of malaria in several regions, primarily affecting the poorest and most vulnerable populations [3]. There is a growing evidence that helminths (or the immune response to helminths) may alter the gut microbiota by favoring specific bacterial communities [3]. A recent study conducted in Ethiopia showed that people infected with *Trichuris trichiura* exhibit lower alpha diversity than uninfected peers [19]. On the other hand, the gut microbiota might influence the host’s immune response towards certain helminths, potentially modulating the severity and outcome of infections [20–22]. Additionally, interactions between *Plasmodium* and helminth infections may alter immune responses and susceptibility of the infected host; thus causing impact on clinical outcome by either worsening (synergism) or reducing (antagonism) the severity of infection and disease [23–25]. Immunomodulation between malaria and STH is a result of two opposing immune response types produced by the two parasites. Malaria-infected hosts mount pro-inflammatory Th1 immune response – dominated by cytokines like interferon-γ (IFN-γ) and tumor necrosis factor-alpha (TNF-α) – to clear *Plasmodium* parasites. In contrast, STH promote a modified anti-inflammatory Th2 immune response which encompasses Transforming growth factor beta (TGF-β), Interleukin-4 (IL-4), IL-5, and IL-10 that favor their survival in the host [26]. It remains poorly understood whether STH immunomodulate malaria responses through altering the gut microbiota. Although coinfections are common, significant gaps remain in our understanding regarding the nature and extent of these interactions, including their directionality and magnitude [27].

Taken together, these findings highlight an incomplete understanding of the impact of malaria on the gut microbiota and vice versa. To assess how different factors could affect gut microbiota composition in malaria-endemic regions, we used a multidimensional approach to investigate malaria-gut microbiome associations within the context of geographic regions, diet, parasitic coinfection and age in Rwanda.

Specifically, this study’s first objective was to analyze the gut microbiota composition by parasitic coinfection, age and geographic regions using 16S rRNA gene amplicon sequencing. Our second objective was to assess the potential role of nutritional habits in observed gut microbial differences.

This study’s findings have the potential to enrich the limited gut microbiota literature in Rwanda. We also shed light on key factors to consider in designing malaria-gut microbiota studies. Most importantly, our work contributes to the WHO’s call for more research to inspire innovations for malaria prevention, control and management in endemic settings [28].

## Materials and methods

### Ethics and consent

Our cross-sectional research project was reviewed and approved (reference number 031/CMHS IRB/2021 issued on the 2^nd^ of February 2021 and reference number 217/CMHS IRB/2022 issued on the 2^nd^ of February 2022) by the Institutional Review Board (IRB) of the College of Medicine and Health Sciences (CMHS) at the University of Rwanda (UR). For both participation and publication of all clinical data, and other data included in this manuscript, written informed consent was obtained from study participants aged 18 and above, and from parents, relatives or guardians of younger participants.

### Study design, participants and samples

**Fig 1:**
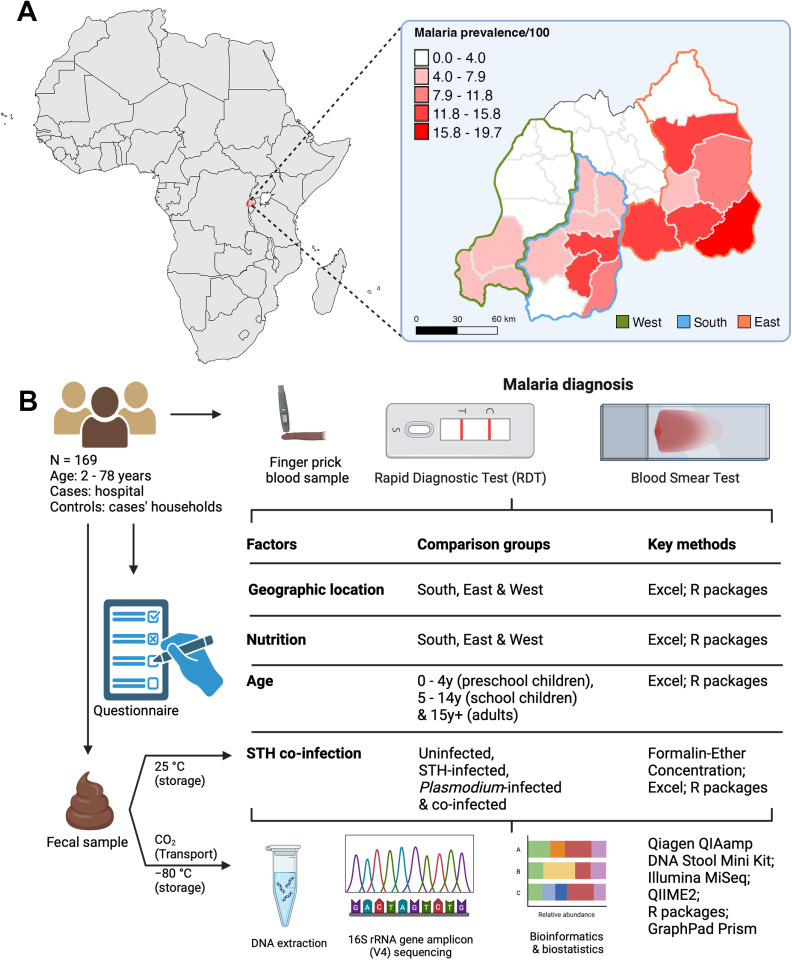
Study area and design. **(A)** Study area showing three malaria-endemic provinces of Rwanda: West, South and East. **(B)** Study design summary including, but not limited to: participant recruitment, sample and data collection, malaria diagnosis, STH screening, key methods of testing, bioinformatics and biostatistical analyses for group comparisons. N, number of participants; STH, soil-transmitted helminths. Created with BioRender.com.

This cross-sectional study was conducted in the Republic of Rwanda. The covered territory included 11 out of 30 districts - belonging to Western, Southern and Eastern provinces - of Rwanda which are classified as malaria-endemic with stable transmission (Fig 1A) [18]. Samples were collected from 169 participants between the 1^st^ of November 2021 and the 30^th^ of September 2022 (Fig 1A). Study participant recruitment took place at either a health facility (cases) or within their household (controls). We used a convenience sampling strategy given our time and budget limitations and in response to the commendable depletion of malaria cases, thanks to robust interventions (e.g., indoor residual spraying) deployed by the government of Rwanda in targeted areas at the time of our study. Cases of malaria-infected participants were found at either district hospitals or health centers. Control participants, who had to be from the same households as cases, were met in their homes on the same day. Study cases were malaria-positive patients found in health facilities within our study area who had not been given antimalarials, antibiotics, and/or antihelminthics in the past two weeks prior to sample collection. Study controls were malaria-negative people, found in the same households of the already recruited cases, preferably with similar age, gender and nutritional habits.

Upon receiving their signed consent forms, a demographic questionnaire was filled for each participant. On the same day, for each participant, a confirmatory (for cases met in health facilities) or screening (for controls met in households) rapid diagnostic test (RDT) was performed, blood smears were prepared for final malaria diagnosis and stool samples were collected for helminth screening and gut bacterial DNA extraction for bacterial 16S rRNA gene sequencing. For malaria diagnosis, blood smear microscopy was considered gold standard, thus its results were considered final to avoid false negative and positive preliminary results of the RDT. That resulted in four comparison groups based on infection status: Uninfected (Neither), *Plasmodium*-infected (Single_P), STH-infected (Single_H) and Co-infected. Age groups (0–4 aged preschool children, 5–14 aged school children and 15 + aged adults) were assigned based on questionnaire-derived qualitative data which was also used to compare nutritional intake and geographic location based on provinces (West, South and East) (Fig 1). Malaria patients identified in health facilities were treated by these same facilities while for other malaria-positive people identified in households (not included in controls), our team provided treatment according to the guidelines of the Ministry of Health effective in the Republic of Rwanda at the time of diagnosis. All samples were collected before any treatment was administered to patients.

### Nutritional questionnaires and body mass index (BMI)

Food intake was evaluated using a 7-day Food Frequency Questionnaire (FFQ) and 24 hour recall questionnaire. FFQ and 24h recall questionnaires were combined to minimize error in recording data related to food items consumed and to enhance complete and accurate food recall [29]. Study participants (both cases and controls) were challenged to recall and list all the foods and drinks they had consumed the day before, using visuals aids provided in “Photographic Food Atlas for Kenyan adolescents (9-14 years)” to approximate the serving sizes of various foods. For the frequency of consumption, four categories were generally available (never or rarely, 1–3 times per week, 4–7 times per week coupled with once or twice per day and 3 times per day). Total quantities of items consumed by study participants were recorded per 7 days. To obtain daily quantities, recorded amounts were divided by 7 before multiplying the result by the number of times the food item was consumed during the week. To translate the quantities of each food consumed in nutrients (macronutrients), we established a Rwanda Food Composition Table (FCT), made from the West Africa FCT 2019 supplemented with the Kenya FCT 2018. Finally, to complete the Rwanda FCT with few items which were not present in the two previously mentioned tables, we used the 7th edition of Belgian FCT 2022. The three FCT were chosen based on the closest proximate in food items, preparation for any given food composition. Thereafter, the Rwanda FCT generated was used to translate each food in macronutrients (protein (g), lipids (g), carbohydrates (g) and fibres (g)) that were then converted into energy intake (kcal). Finally, the nutrition analysis was undertaken using GraphPad Prism version 10.0.0 for Windows, GraphPad Software, Boston, Massachusetts USA, www.graphpad.com.

The BMI was calculated by dividing the participant’s weight (in kg) by the square of their height (in meters) and was expressed in kg/m². As recommended by the WHO, sex- and age-specific BMI-for-age Z-scores were used for participants aged 2–19 [30–32].

### Rapid test and blood film malaria diagnosis

Trained laboratory technicians used sterile, single-use lancets to draw finger-prick blood used to perform a malaria RDT for on-site immediate confirmation or screening. Additionally, thick and thin blood films on glass slides for the identification of *Plasmodium* species and the determination of parasite density (parasitemia) at the National Reference Laboratory. Prepared blood smears were stained with Giemsa as previously described [33]. A compound microscope was used to determine *Plasmodium* species and parasite density on stained smears fully covered with immersion oil type A. Following complete positive diagnosis combined with questionnaire generated data, participants were classified according to clinical manifestations as: having ‘severe’ malaria (cerebral malaria, respiratory distress, severe malarial anemia, malaria with complicated seizures, and prostration); having ‘mild’ malaria (fever and flu-like illness, including one or all of the following symptoms: shaking chills, headache, muscle aches, tiredness, nausea, vomiting, and diarrhea) and having ‘asymptomatic’ malaria (no symptom). Control participants with a laboratory negative diagnosis and with no symptoms were classified as ‘uninfected’ with malaria.

### Stool samples and Soil-Transmitted Helminths (STH) screening

After technicians had collected malaria blood samples, adult participants or guardians were given clear instructions to collect stool samples on single-use aluminum plates. Upon reception from the participant/guardian, stool samples were divided into two parts. Part one was transferred into a formalin-ether container for helminth screening. Part two was divided into three aliquots (5mL per tube), and kept under anaerobic conditions by GasPak EZ Anaerobe Pouch System with Indicator, 20 (BD Diagnostic Systems, USA) for a maximum of 24 hours during sample transport. Both parts were transported the same day from field to the laboratory where part one was kept at room temperature until STH screening and part two frozen (−80 °C) until bacterial DNA extraction. STH screening results were recorded as negative or positive plus causative species (i.e., *Trichuris trichiura*, *Ascaris lumbricoides*, and hookworm : *Ancylostoma duodenale* and or *Necator americanus*). All STH-positive participants received screening results and antihelmintic treatment through community health worker channels in maximum two weeks after sample collection. Treatment followed the guidelines of the Ministry of Health effective in the Republic of Rwanda at the time of diagnosis.

### Stool bacterial DNA extraction, sequencing and gut microbiota composition analysis

Bacterial DNA extracted from fecal samples was sequenced for gut microbiota composition analysis. Samples were kept frozen at −80°C until DNA extraction. The extraction of metagenomic DNA was carried out using QIAamp Fast DNA Stool Mini Kit (Qiagen, USA) according to the manufacturer’s instructions with the addition of a homogenization step by bead-beating. DNA purity (A260/A280) and concentration were determined using a NanoDrop2000 (Thermo Fisher Scientific, USA).

Samples were diluted in TE buffer to a concentration of 20ng/µl and sent to MrDNA (www.mrdnalab.com; Shallowater, TX, USA) for sequencing. The V4 region of bacterial 16S rRNA gene was amplified using the primers 515F (5’-GTGYCAGCMGCCGCGGTAA-3’) and 806R (5’-GGACTACNVGGGTWTCTAAT-3’) [34]. Purified amplicons were sequenced using the Illumina MiSeq platform (2x250 bp PE) according to the manufacturer’s guidelines, followed by data demultiplexing.

The analysis of demultiplexed paired-end FASTQ files provide by MrDNA was performed using QIIME2 (version 2024.10-amplicon) [35] on an Apple M2 Max system. Primer sequences were trimmed, and the quality of the sequences were assessed through visualization of interactive quality plots. Denoising and merging of paired-end reads were conducted using the QIIME2 DADA2 plugin, generating amplicon sequence variants (ASVs) [36]. Truncation and trimming parameters were optimized based on the quality plots to maintain the highest sequence quality (quality score of 37 or more for the 25th, 50th, 75th, and 91st percentiles). To enhance the reliability of downstream analyses, low-abundance features (<5 counts across samples) and poor-quality samples (<40000 sequence reads) were filtered out. Features were clustered de novo based on sequence similarity using the QIIME2 VSEARCH cluster plugin with a 99% identity threshold. This clustering step reduced computational complexity and enhanced the accuracy of subsequent chimera detection. Chimeric sequences were identified and removed using the UCHIME [37] algorithm within the VSEARCH plugin [38]. Taxonomic classification was performed using a pre-trained classifier based on the SILVA v138 reference database [39]. Finally, we filtered out chimeric sequences and unwanted sequences (Eukaryotes, chloroplasts, mitochondria) (S1 Table). For phylogenetic analyses, a phylogenetic tree was constructed to enable diversity assessments. This step involved sequence alignment using MAFFT [40] and tree building with FastTree [41]. Alpha diversity metrics (Observed features, Shannon entropy, Faith’s phylogenetic diversity, Chao1 and Pielou evenness) were calculated using rarefied feature tables (threshold at 47000 reads) (S1 Fig) [42–45]. Beta diversity metrics (Bray-Curtis – used for PCoA visualization, Weighted Unifrac, Unweighted Unifrac and Jaccard) were also computed to examine community composition differences between samples [46–48]. To identify differentially abundant taxa across experimental conditions, Analysis of Composition of Microbiome with Bias Correction (ANCOM-BC) was employed [49]. This analysis was conducted at multiple taxonomic levels, including phylum, family, and genus. Finally, the processed data were exported for further statistical analysis and qza files generated in QIIME2 were used for visualization in R (version 4.4.1, R Core Team, 2024) [50]. With the package qiime2R, we imported QIIME2-generated distances into R to produce principal coordinate analysis (PCoA) beta diversity plots using ggplot2, glue, tidyverse, ggrepel, dplyr and ggExtra packages [51–56]. GraphPad Prism (version 10) was used to create alpha diversity figures using QIIME2-generated metrics [57].

### Statistical analysis

Statistical significance of group comparisons was analyzed by QIIME2 (version 2024.10-amplicon), GraphPad Prism (version 10) and R (version 4.4.1). In GraphPad, we used the ROUT method to remove outliers (Q = 1%). Next, for alpha diversity and nutritional data, we used ANOVA (analysis of variance) or Kruskal Wallis tests if data were normally distributed or not (by Shapiro-Wilk test) respectively. Follow-up multiple pairwise comparisons were determined by Dunn’s test (alpha diversity) and post hoc Bonferroni (nutrition). QIIME2’s PERMDISP test was performed to determine sample homogeneity (non-significant result), before running PERMANOVA and pairwise PERMANOVA (999 permutations for both) for beta diversity. Group comparisons were considered significantly different at *p* < 0.05.

For further efficient exploratory purposes, Spearman’s correlations were tested to assess associations between differentially abundant, fully identified bacterial genera (identified in the ANCOM-BC analysis) and other key variables. The latter included age, macronutrient values, *Plasmodium* parasitemia and alpha-diversity metrics. Correlations were computed in R using the package ‘Psych’ (version 2.3.6) with FDR multiple testing correction [58]. The corresponding r-score, p-value and adjusted p-values were listed in a table (S2 Table). A correlogram representing the correlation and statistical significance levels was generated using the R package ‘Corrplot’ (version 0.92) [59]. Overall, specific statistical tests and significance cutoffs are described in figure legends. Group comparisons were considered significantly different at p-value/adjusted *p* < 0.05.

## Results

### Study design

This cross-sectional study was designed to assess the gut microbiota composition within the context of geographic regions, nutrition, parasitic coinfection, and age in malaria-endemic regions of Rwanda (Fig 1A). Therefore, we recruited participants from Rwanda’s Western, Southern and Eastern provinces. Demographic data, blood and fecal samples were collected from 169 participants (85 females and 84 males) aged between 2–78 years. Malaria diagnosis was followed by soil-transmitted helminth (STH) screening which enabled us to make four comparison groups based on infection status: Uninfected (Neither), *Plasmodium*-infected (Single_P), STH-infected (Single_H) and Coinfected (Fig 1B). Socio-demographic characteristics of the study population are summarized in Table 1.

**Table 1:** Characteristics of the study population.

Characteristics: units	Provinces				
	Overall	East	West	South	p-value
Participants: n	169	73	50	46	
Females: n (%)	85 (50.3)	38 (52)	26 (52)	21 (45.6)	
Age: median (range)	16 (2-78)	16 (2-78)	15 (4-73)	22.5 (2-64)	0.15 (Kruskal-Wallis)
BMI: mean±SD kg/msq	19.1 ± 3.8	19.6 ± 3.9	18 ± 3.7	19.6 ± 3.7	0.03* (Kruskal-Wallis)
Uninfected: n (%)	37 (21.9)	24 (32.9)	4 (8)	9 (19.5)	
Only Plasmodium-infected: n (%)	129 (76.3)	48 (65.7)	45 (90)	36 (78.3)	
Plasmodium parasitemia: mean±SD parasites/ul	16,058 ± 32,330	23,818 ± 38,891	17,376 ± 33,762	6,495 ± 18,401	0.18 (Kruskal-Wallis)
Plasmodium-uninfected: n (%)	40 (23.7)	25 (34.2)	5 (10)	10 (21.7)	
Asymptomatic Plasmodium-infected: n (%)	2 (1.2)	2 (2.7)	0 (0.0)	0 (0.0)	
Mild Plasmodium-infected: n (%)	121 (71.6)	40 (54.8)	45 (90)	36 (78.3)	
Severe Plasmodium-infected: n (%)	6 (3.5)	6 (8.2)	0 (0.0)	0 (0.0)	
Coinfection: n (%)	14 (10.8)	8 (16.7)	4 (8.9)	2 (5.5)	
Only STH-infected: n (%)	3 (7.5)	1(4)	1(2)	1(10)	
Total STH-infected: n (%)	17 (10)	9 (12.3)	5 (10)	3 (6.5)	
AL-infected: n (%)	10 (5.9)	6 (8.2)	1 (2.2)	3 (6)	
TT-infected: n (%)	3 (1.8)	1 (1.4)	1 (2)	1 (2.2)	
HW-infected: n (%)	2 (1.2)	1 (1.4)	1 (2)	0 (0.00)	
Double (TT and AL) STH-infected: n (%)	1 (0.6)	0 (0.00)	1 (2.8)	0 (0.00)	
Triple (TT, AL and HW) ST- infected: n (%)	1 (0.6)	1 (1.4)	0 (0.00)	0 (0.00)	

Study participants were recruited from the Western, Southern and Eastern provinces of Rwanda. n, number; %, percent; STH, Soil-Transmitted Helminths; TT, *Trichuris trichiura*; AL, *Ascaris lumbricoides*; HW, Hookworm (*Ancylostoma duodenale* and/or *Necator americanus*). SD, standard deviation; Statistical significance:

* : *p* < 0.05.

The bacterial 16S rRNA gene (V4 region) amplicon sequencing carried out on 169 samples yielded 126,748,404 paired-end reads (S1 Table). After quality control, denoising and filtering, we obtained 3,166 amplicon sequence variants (ASVs) with an average of 387,370 reads per sample across the dataset.

### Unique gut microbiota profiles observed in the Western province

**Fig 2:**
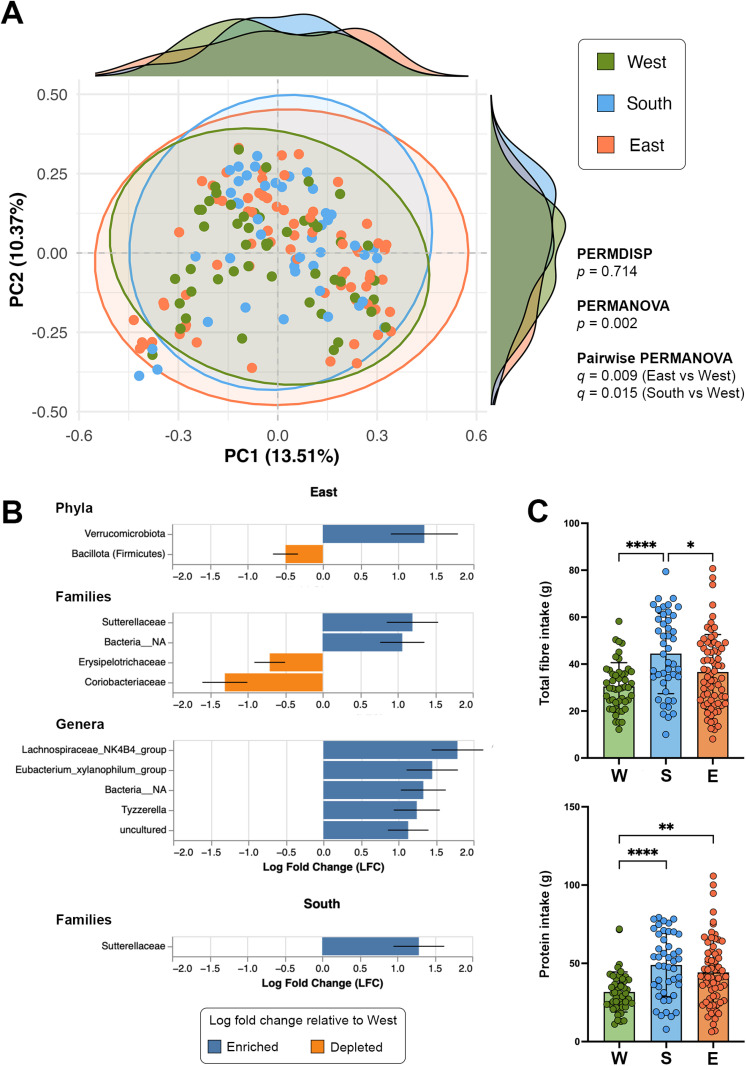
Panels show results of gut microbiota and nutritional intake analyses by provinces. **(A)** Beta diversity analyses, visualized by a PCoA, reveal statistically significant differences between the West and both the East and the South; **(B)** ANCOM-BC analysis results show differentially abundant taxa between the West and the East at phylum, family and genus levels while only one family was differentially abundant between the West and the South. **(C)** Nutritional intake analyses show decreased levels of both total fibre and proteins in the West compared to the East and South. For panels A and C, each point represents an individual sample. For panel C, box plots represent the mean with standard deviation (SD) of the samples. Statistical significance: *: *p* < 0.05, **: *p* < 0.01, ***: *p* < 0.001, ****: *p* < 0.0001.

While all alpha diversity metrics tested showed non-significant differences, beta diversity analyses demonstrated notable differences between provinces at genus level. Using Bray-Curtis distances, a Principal Coordinates Analysis (PCoA) was performed to test and visualize beta-diversity between provinces. PC1 and PC2 principal coordinates’ explained variance is 13.51% and 10.37% respectively. A non-significant PERMDISP test result (*p* > 0.05) excluded the possibility of a sample dispersion bias, allowing us to perform a PERMANOVA test which confirmed significant differences between provinces (*p* < 0.05). The pattern observed in the central confidence ellipses and the marginal density plots indicates the degree of variability between provinces; with samples from the West clustering relatively together compared to the South and the East (Fig 2A).

By pairwise comparisons, significant differences were observed between the East and West regions using Jaccard (pseudo-F = 1.809, *q* = 0.024), Unweighted UniFrac (pseudo-F = 2.530, *q* = 0.015), and Bray-Curtis (pseudo-F = 2.431, *q* = 0.0045). However, Weighted UniFrac did not reach statistical significance (pseudo-F = 1.956, *q* = 0.1).

Similarly, the South and West regions differed significantly with Jaccard (pseudo-F = 1.524, *q* = 0.0405), Unweighted UniFrac (pseudo-F = 1.896, *q* = 0.0405), and Bray-Curtis (pseudo-F = 2.010, *q* = 0.0045), while Weighted UniFrac did not pass the statistical threshold (pseudo-F = 2.047, *q* = 0.1). In contrast, comparisons between the East and South regions did not reveal significant differences across any metric.

The Analysis of Compositions of Microbiomes with Bias Correction (ANCOM-BC) returned differentially abundant taxa between the West and the East at phylum, family and genus levels while only one family was differentially abundant between the West and the South. At phylum level, *Verrucomicrobiota* were enriched while *Bacillota* (formerly called ‘*Firmicutes*’) were depleted in the East compared to the West. At family level, *Sutterellaceae* and unidentified (Bacteria_NA) were enriched while *Erysipelotrichaceae* and *Coriobacteriaceae* were depleted in the East versus the West. Out of five, three fully identified genera (*Lachnospiraceae*_NK4B4_group, *Eubacterium_xylanophilum*_group and *Tyzzerella*) were enriched in the East while only one family (*Sutterellaceae*) was enriched in the South (Fig 2B).

### Generally poor nutritional intake observed in the Western province

Nutritional intake analyses revealed differences between the Western, Southern and Eastern provinces. The three groups were compared by the values of total fibre, total energy, proteins, carbohydrate and total lipid intake. As summarized in Table 2, overall significant differences were reported between fibre intake (*p <* 0.001) and protein intake (*p* < 0.002). Pairwise comparisons revealed that total fibre intake were significantly lower in the West compared to the South (*q* < 0.0001), while a tendency was observed compared to East (*q* = 0.07) by one-way ANOVA. Furthermore, we found that protein intake was significantly lower in the West compared to both the South (*q* < 0.0001) and the East (*q* < 0.002) (Fig 2C). For all the other nutritional intake types measured, we observed lower values in the West compared to other two provinces, but differences were not statistically significant (Table 2). Taken together, these findings paint a picture that distinguishes the Western province from both the Eastern and Southern provinces.

**Table 2:** Nutritional intake values by provinces.

Nutritional intake type	Provinces			
	West (n = 50)	East (n = 72)	South (n = 46)	p-value (ANOVA)
Total fibre intake in grams (Mean± SD)	31.4** ± **12.1	37.4** ± **18.3	44.7** ± **17.1	< 0.001***
Total Energy intake in kilocalories (Mean±SD)	1406.3** ± **449.9	1535.5** ± **858.9	1667.9** ± **537.0	0.16
Proteins intake in grams (Mean±SD)	34.1** ± **16.8	46.9** ± **32.5	49.1** ± **19.9	0.006**
Carbohydrates intake in grams (Mean±SD)	243.8** ± **71.8	247.3** ± **95.5	280.65** ± **94.5	0.08
Lipids intake in grams (Mean±SD)	25.8** ± **12.1	30.6** ± **49.7	27** ± **11.1	0.71

The table summarizes nutritional intake values for the three provinces: West, East and South. n, number of participants; SD, standard deviation; Statistical significance:

* : *p* < 0.05,

** : *p* < 0.01,

*** : *p* < 0.001.

### Specific gut microbiome profiles were associated with age

**Fig 3:**
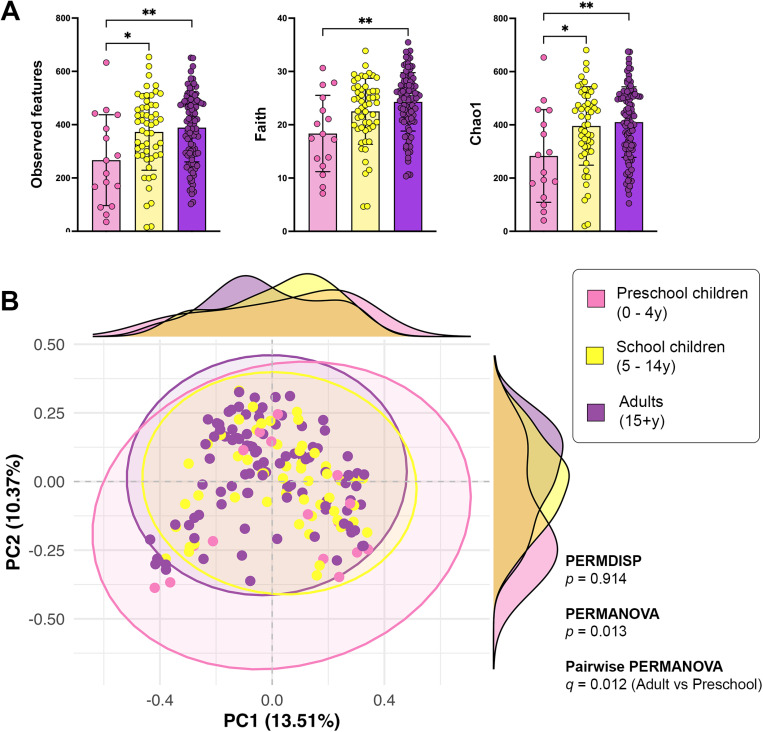
Alpha and beta diversity analyses by age-groups. **(A)** Observed features, Faith phylogenetic diversity and Chao1 metrics revealed significantly lower alpha diversity in preschool children than in adults and school children. **(B)** By beta diversity analyses, the adults group is statistically different from the preschool group. For both panels, each point represents an individual sample. Box plots represent the median (bar), interquartile range (box), and 95% confidence interval (whiskers). Statistical significance: *: *p* < 0.05, **: *p* < 0.01.

Alpha diversity analysis contributed to most of the differences observed in the gut microbiota profiles between age groups with a minor contrast observed in beta diversity. Additionally, ANCOM-BC returned differentially abundant taxa between specific age groups. Alpha diversity analysis showed statistically significant differences between the preschool children group and both school children and adults groups. Observed features showed that preschool children exhibited significantly lower microbial diversity compared to school children (H = 5.571, *q* = 0.027) and adults (H = 8.488, *q* = 0.011) (Fig 3A). No significant differences were observed between school children and adults (*p* = 0.594, *q* = 0.594). Shannon diversity index analyses indicated significant differences between preschool children and adults (H = 5.379, *p* = 0.020, *q* = 0.061), though these differences did not persist across all comparisons after correction for multiple testing. Faith’s phylogenetic diversity showed that preschool children differed significantly from adults (H = 9.086, *q* = 0.008), with differences between preschool and school children approaching significance (H = 4.428, *p* = 0.035, *q* = 0.053) (Fig 3A). Evenness analyses revealed no significant differences in microbial community evenness among age or province groups (all *q* > 0.1).

Beta-diversity analysis was conducted and a PCoA plot was generated to visualize differences between the adults, school children, and preschool children age groups using Bray-Curtis distances. Results of the PERMDISP and PERMANOVA tests allowed us to perform a pairwise PERMANOVA test whose results confirmed differences in beta-diversity between preschool children and adults (*p* < 0.05) (Fig 3B). This suggests that the microbial community composition differs notably between these two age groups. The marginal density plots show distinct distributions for the three groups, particularly between preschool children and adults. However, overlap in the confidence ellipses and density plots indicates minimal degree of variability within groups, particularly for school children, which appear intermediate between preschool children and adults (Fig 3B).

**Fig 4:**
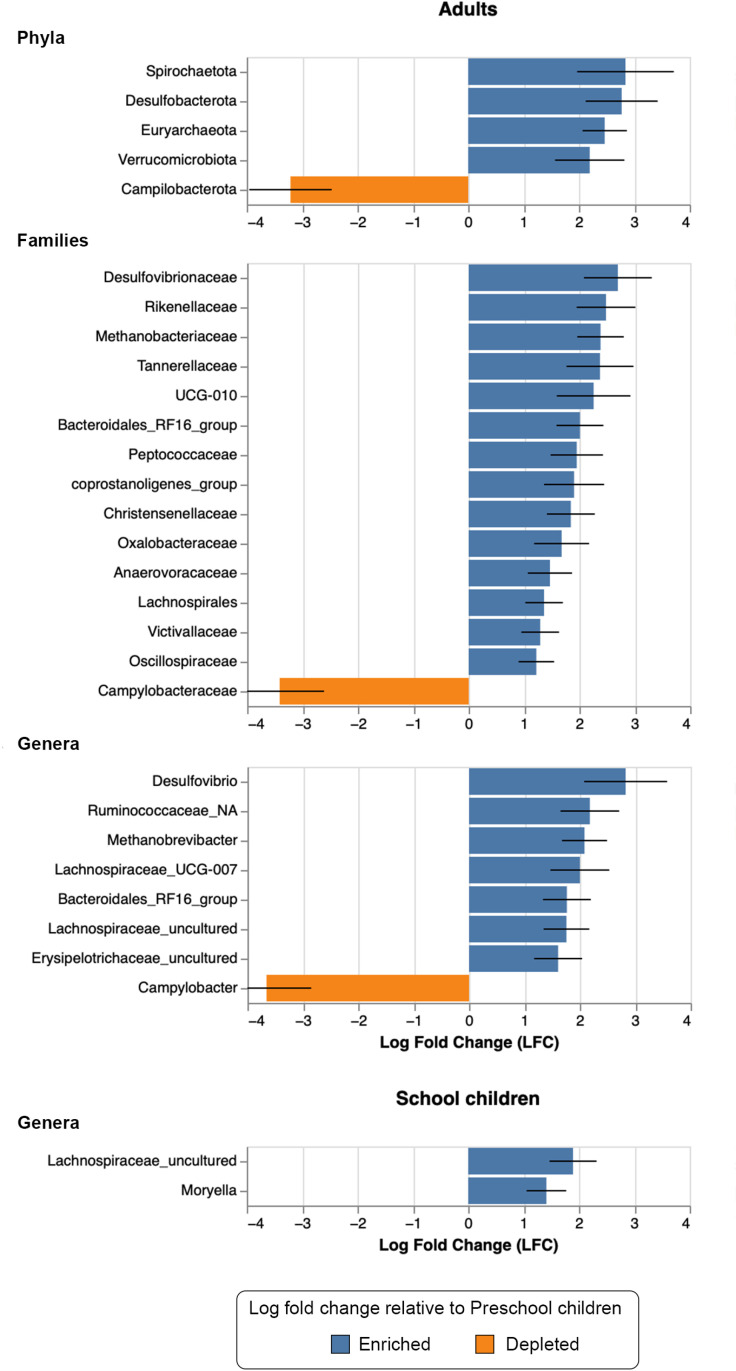
ANCOM-BC differential abundance analysis by age-groups. Results show differentially abundant taxa between adults and preschool children at phylum, family and genus levels while only two genera were differentially abundant between school children and preschool ones.

ANCOM-BC analysis of differential taxa relative to preschool children revealed 5 phyla, 15 families and 8 genera unique to adults whereas only two genera were unique to school children. While majority of the taxa were enriched, phylum *Campilobacterota*, family *Campylobacteraceae* and genus *Campylobacter* were depleted in adults relative to preschool children. The same analysis returned two genera (*Lachnospiraceae*_uncultured and *Moryella*) uniquely enriched in school children relative to preschool ones (Fig 4). This observation implies that major and minor differences were observed in the adults and school children, respectively, in comparison to preschool children.

### No association observed between infection status and gut microbiota profiles

Malaria infection alone or its coinfection with STH were not associated with specific gut microbiota signatures. Malaria was confirmed in 129 participants while 40 tested negative. Average *Plasmodium* parasitemia (parasites per microliter of blood) per province was 23,818 in the East; 17,376 in the West and 6,495 in the South (Table 1). Differences were not statistically significant between provinces (*p* > 0.05). However, observed variations may be explained by other factors. For example, all six (6) severe cases reported in this study were from the East which exhibited higher average parasitemia compared to the other two provinces. Notably, we could not compare malaria diagnosis results based on severity because, apart from mild cases whose distribution was not significantly different between provinces, asymptomatic and severe cases were detected in the East only (Table 1).

STH screening results revealed that 17 participants were infected with STH which represents 10% of the studied population (n = 169). Affecting 5.91% of all the studied population, *Ascaris lumbricoides* (AL) was the most prevalent STH species, and it was found to affect more people in the East than in both the West and the South combined. The second most prevalent STH species was *Trichuris trichiura* (TT) followed by Hookworm (HW) - *Ancylostoma duodenale* and/or *Necator americanus*. Double (TT and AL) and triple (TT, AL and HW) STH infections were rare. Only 3 participants were infected with STH alone in our study (Table 1).

Coinfection affected 14 participants. Among provinces, coinfection prevalence was 16.7%, 8.9% and 5.5% in the Eastern, Western and Southern provinces respectively (Table 1).

Gut microbiota analysis by infection group revealed no statistically significant differences among the four compared groups: Coinfection, Uninfected, *Plasmodium*-infected and STH-infected. Alpha diversity analyses results were statistically non-significant between groups by all metrics used in this study (S2 Fig). Beta diversity analysis using Bray Curtis distances were also non-significant (*p* = 0.992 by PERMANOVA) (S2 Fig).

### A multifactorial analysis yielded significant positive correlations

**Fig 5:**
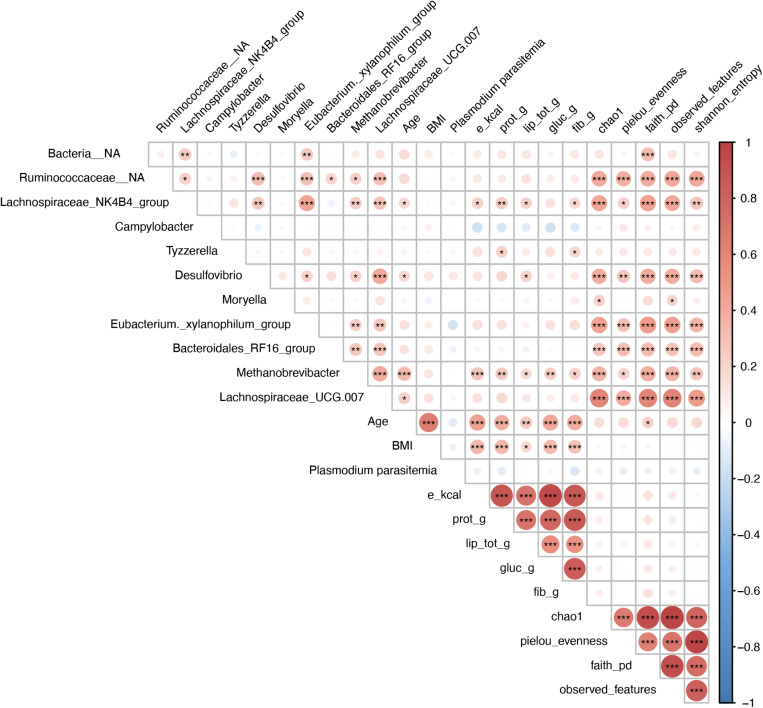
Spearman’s correlations between key differentially abundant bacterial genera (by ANCOM-BC), nutritional intake values, alpha diversity metrics and key metadata variables (age, BMI and *Plasmodium* parasitemia). The Correlogram shows positive (red) and negative (blue) correlation with, where applicable, statistical significance. Statistical significance: *: *p* < 0.05, **: *p* < 0.01, ***: *p* < 0.001. lip_tot_g, lipid (total) intake in grams; gluc_g, glucose/carbohydrates intake in grams; fib_g, fibre intake in grams; prot_g, protein intake in grams and e_kcal, energy intake in kilocalories.

Correlational analysis of multiple factors showed important relationships between differentially abundant genera (identified in the ANCOM-BC analysis), alpha diversity metrics, nutritional intake values, age, and BMI (Fig 5). Differentially abundant genera showed remarkable positive correlation with alpha diversity metrics. Significant positive correlations were observed between six genera (*Methanobrevibacter*, *Desulfovibrio, Eubacterium_xylanophilum*_group, *Bacteroidales*_RF16_group and *Ruminococcaceae*_NA, *Lachnospiraceae*_UCG.007) and all five alpha diversity metrics (Chao1, Pielou evenness, Faith’s pd, observed features and Shannon entropy) while Bacteria_NA only showed correlation with Faith’s pd and *Moryella* with Chao1 and observed features (Fig 5).

Two genera, *Campylobacter* and *Tyzzerella*, did not exhibit significant correlations with any metric. These results suggest that specific bacterial genera play essential roles in determining intestinal community differences within samples. Age and BMI significantly correlated with nutritional intake types (energy, proteins, lipids, carbohydrates and fibres). Age was also positively correlated to four bacterial genera: *Lachnospiraceae*_NK4B4_group, *Desulfovibrio*, *Methanobrevibacter* and *Lachnospiraceae*_UCG.007. This highlights the evolution of the gut microbiota with age. *Plasmodium* parasitemia showed no statistically significant correlation across all other factors. This observation reinforces the lack of association reported between malaria infection and the gut microbiota composition in our study. All nutritional intake types were significantly correlated with one or multiple of the following differentially abundant genera: *Lachnospiraceae*_NK4B4_group, *Tyzzerella* and *Methanobrevibacter*. Such observation shows the association between nutritional intake and the gut microbiota composition. Interestingly, *Desulfovibrio* was the only genus that correlated significantly with only one nutritional component (total lipid intake), suggesting a potentially specific relationship between these two variables (Fig 5).

## Discussion

In this study, we demonstrate significant differences in the gut microbiota composition of people living in three different malaria-endemic provinces of Rwanda. Our multifactorial analysis allowed us to assess the contribution of different factors likely to influence the gut microbiota composition such as the host geographic location, nutritional intake, parasitic coinfection and age. In our settings, geographic location and host age showed differences in the gut microbial composition differences whereas infection status (including malaria-STH coinfection) showed non-significant results.

Indeed, we discovered a geographic variation associated with a different beta-diversity of the gut microbiota in the Western province of Rwanda. These findings are consistent with the results of a study conducted by Yooseph *et al*. (2015) in Mali which identified significant differences in the gut microbiota composition between the Malian cohort and cohorts from Malawi and other countries around the world [8]. The relationship between geography and microbiome profiles has also been shown by studies conducted in Tanzania and Botswana as well as in South Africa [60–62]. Our study stands out by using primary data to identify gut microbial differences among regions within one, small territory with relatively less host genetic variations and taking into consideration the host age, infection/coinfection status and nutritional habits. Additionally, the Western province of Rwanda presents unique environmental aspects such as being separated from the rest of the country by the Nyungwe Forest National Park which was recently added to UNESCO’s world heritage [63]. Consequently, that makes the West particularly disconnected and relatively more rural compared to the South and the East which are directly connected with Kigali – the capital city of Rwanda.

Nutrition plays a fundamental role in shaping the gut microbiota composition. Evolutionarily, it has been shown that dietary intake is directly linked to gut microbial diversity of mammals including humans [64]. In their review, Wiertsema *et al*. discussed the role of nutrition in modulating the effects of the gut bacterial communities on infections such as malaria [65]. In the present study, nutritional analyses revealed that, regardless of infection status, a low-fibre intake may explain the differentially unique gut microbial beta diversity observed in the Western Province compared with the East and the South. Indeed, total fibre intake was 20–30% lower in the Western than in the two other provinces. In addition, compared with Southerners and Easterners, participants from the Western province were characterized by significantly lower BMI levels and lower total energy intake, although not statistically different. Interestingly, the Spearman’s correlation analysis showed that the BMI significantly correlated mostly with all nutritional intake types (energy, proteins, lipids, carbohydrates and fibres) followed by selected bacterial genera. Hence, compared to other studies, our findings confirm the association between low dietary fibre intake and lower gut microbiota diversity in human cohorts. However, there were other studies which reported statistically non-significant differences by alpha and beta diversity analyses [66,67]. Therefore, the impact of fibre intake is commonly seen as a factor that may influence the diversity of gut microbiota [68], although not alone nor always [69].

In Africa, low dietary fibre intake is often reported as a characteristic of common forms of malnutrition such as Kwashiorkor [70]. Thus, in our case, lower fibre among other generally low nutritional intake types may be linked to the malnutrition and food insecurity reported in the Western province by the Rwandan national institute of statistics [71]. Consistent with our results, a study conducted in Bangladesh communicated that malnutrition impaired the maturation and diversification of the gut microbiota [72]. Moreover, lower gut bacterial diversity can be associated with a less varied diet in cultures. For example, this was observed in the nomadic, pastoral Fulani people living in rural settings compared to Jawara ethnicity who, despite dwelling in urban Nigeria, consume fibrous and fermented foods in addition to processed diets [73]. Taken together, the findings of this and previous studies argue that low-fibre nutrition can lead to a comparatively different gut microbiota composition like we observed in the Western province of Rwanda. Exposure to a large variety of environmental microbes associated with a high-fibre diet could increase potentially beneficial bacteria and enrich microbial diversity. A reduction in the gut microbial richness, because of low fibre intake, has been associated with poor health outcomes [74–76]. Hence, our findings call for further investigations in this regard.

The differences observed in the gut microbiota composition were not associated with infection status. Neither beta diversity nor alpha diversity analyses revealed any statistical differences associated with infection status. Consequently, differential abundance as well as correlational analyses revealed no relationship between gut bacteria and infection status (i.e., *Plasmodium* parasitemia). Similar findings were reported by Yooseph *et al.* in their study which failed to observe an association between gut microbiome composition and febrile malaria after *Plasmodium* had reached blood stage of infection [8]. Another study conducted in Kenya reported that only the number of malaria episodes and antimalarial treatment explained differences, although minimal, in microbial profiles [12]. In the context of STH-*Plasmodium* coinfection, *P. vivax* – microbiome association was shown in a Colombian study [10] and higher levels of *Lactobacilli* were reported among the microbial communities of *P. vivax*–infected people in India [9]. These findings inform us that, in general, apart from mild infection with *P. vivax,* available studies about *Plasmodium* and STH parasites have shown limited potential to modify the human gut microbiota. These discrepancies could be explained by differences in the genetics of the host, parasite (*P. vivax*) or even vectors plus environmental factors (e.g., geographic location) which are obviously different between Africans and Americans or Asians. In particular, Easton *et al.* [10] speculated that geography could be a possible reason for non-differential gut microbiota composition between STH-infected and -uninfected groups in Colombia whereas *T. trichiura* infection was associated with greater microbial diversity among infected individuals in Malaysia.

We reported associations between specific gut microbiota profiles and age groups with preschool children showing significantly lower alpha diversity than school children and adult. Our ANCOM-BC differential abundance analyses showed that, relative to preschool children group, genus *Campylobacter* was depleted in the adults group while two genera (*Lachnospiraceae*_uncultured and *Moryella*) were enriched in school children group. Furthermore, relationship assessment by Spearman’s correlation shed light on significant positive relationships between age and specific bacterial taxa as well as alpha diversity metrics. In accordance with the present results, a study conducted in Mali reported that age may be a stronger predictor of gut microbiota composition than *P. falciparum* infection status [8]. Consistently, Palmer *et al*. showed that clearly noticeable changes take place during preschool (below 5) stage, arguably due to the shift from breastfeeding to consuming solid foods [77]. Furthermore, the alpha diversity has consistently been demonstrated as the right measurement of the effects of age on the gut microbiome [78].

Amongst the differentially abundant bacterial genera (identified in the ANCOM-BC analysis), three genera belonging to the *Lachnospiracea* family (*Tyzzerella, Lachnospiraceae NK4B4 and Eubacterium xylanophilum* group*)* were increased in the Eastern province relatively to the West. *Lachnospiraceae* as a family is an abundant component of the human digestive tract and has been involved in the production of butyrate from dietary fibres [79]. This has been shown specifically for these three genera in *in vitro* and animal models of fibre supplementation [80]. This is in line with our results, as *Tyzzerella, Lachnospiraceae NK4B4 and Eubacterium xylanophilum* group were depleted in the Western province, which had a lower fibre intake. Genus *Desulfovibrio* was enriched in adults relative to preschool children. Although one of the most prevalent genera of the human microbiota, both beneficial and detrimental associations with health and diseases were described for this genus. While associations with a low-fat diet and exercises in humans or a protective effect on non-alcoholic fatty liver disease in a murine model have been observed, its increased abundance has been associated with intestinal and extra-intestinal diseases in clinical and pre-clinical settings (e.g., cancer, metabolic diseases and Parkinson’s disease) [81]. In our study *Desulfovibrio* was positively correlated with total lipid intake which is similar to an observation in a mouse model of high fat diet [82]. *Bacteroidales RF16* group, was another genus enriched in the adult group in our study. While little information is available about this genus in human cohorts, it has been linked to fibre consumption in ruminants [83]. This association was not found in our study, suggesting that further studies are needed to understand how nutrition and this genus are associated. Finally, *Campylobacter* was enriched in preschool children when compared to adults in our study. This bacterial genus is the most common cause of gastroenteritis in the world with children being particularly affected [84]. Overall, associations between the identified genera, with the exception of *Campylobacter,* and health and diseases are only at the level of association and no causality has been established yet which warrants future investigations.

This study presents limitations as to the generalizability of its findings. One is related to low STH infection prevalence and the limited number of cases of extreme malaria severity (asymptomatic and severe). Another limitation is related to the recall bias experienced when answering the food frequency questionnaire [85]. Also, we believe that longitudinal studies could generate more insights needed to assess how age and diet influence microbiota in malaria-endemic regions. In addition, instead of the basic 16S rRNA gene, metagenomic sequencing methods would certainly add insights regarding gut microbial functions. Therefore, we recommend more studies to inform our understanding of associations between gut microbiota and malaria as well as other infections in various demographic groups and geographic settings. Finally, given the role of nutrition, it would be of interest to link the microbiota composition and potential microbial functions with specific micronutrients.

In summary, our results demonstrate that microbial diversity is significantly influenced by age, particularly in preschool children, who exhibit distinct microbial communities compared to adults. Geographic differences, though present, were primarily observed between East and West regions, while the Southern region displayed less pronounced variability. Nutrition intake analyses added another layer of contrast between geographic regions with the West showing lower intake of fibre and proteins compared to the South and East. We observed no link between infection groups and the gut microbiota composition. Finally, a multifactorial analysis revealed significant correlations between differentially abundant genera, alpha diversity metrics, age, BMI and nutritional intake.

## Conclusions

In conclusion, our study contributed to the limited body of literature about the gut microbiota composition in Africa and more specifically in Rwanda. Using a multifactorial approach, we were able to demonstrate that unique microbial profiles observed in the Western province of Rwanda could be linked to a low-fibre nutrition intake. However, unlike age, infection status was not associated with significant differences in the composition of the gut microbiota of the studied population. This study’s findings have the potential to pave the way for research-driven alternative innovations (i.e., microbiota-modulating diets) to control malaria in endemic settings.

## Supporting information

S1 Fig Rarefraction curves of Faith’s phylogenetic diversity and observed features for all samples. (TIF)

S2 Fig Alpha and beta diversity analyses by infection groups.
(TIF)

S1 Table The number of reads filtered out at each step of the DADA2 pipeline.
(XLSX)

S2 Table Significance of correlations after p-value adjustment.
(XLSX)

## References

[1] Dorlo TPC, Fernández C, Troye-Blomberg M, de Vries PJ, Boraschi D, Mbacham WF. Poverty-related diseases college: a virtual african-european network to build research capacity. BMJ Glob Health. 2016;1(1):e000032. doi: 10.1136/bmjgh-2016-000032 28588923 PMC5321328

[2] World malaria report 2023. Geneva: World Health Organization; 2023. Licence: CC BY-NC-SA 3.0 IGO.

[3] Mutoni JD, Coutelier J-P, Rujeni N, Mutesa L, Cani PD. Possible interactions between malaria, helminthiases and the gut microbiota: a short review. Microorganisms. 2022;10(4):721. doi: 10.3390/microorganisms10040721 35456772 PMC9025727

[4] Wang B, Yao M, Lv L, Ling Z, Li L. The human microbiota in health and disease. Engineering. 2017;3(1):71–82. doi: 10.1016/j.eng.2017.01.008

[5] Yilmaz B, Portugal S, Tran TM, Gozzelino R, Ramos S, Gomes J, et al. Gut microbiota elicits a protective immune response against malaria transmission. Cell. 2014;159(6):1277–89. doi: 10.1016/j.cell.2014.10.053 25480293 PMC4261137

[6] Mandal RK, Mandal A, Denny JE, Namazii R, John CC, Schmidt NW. Gut bacteroides act in a microbial consortium to cause susceptibility to severe malaria. Nat Commun. 2023;14(1):6465. doi: 10.1038/s41467-023-42235-0 37833304 PMC10575898

[7] Mandal RK, Schmidt NW. Mechanistic insights into the interaction between the host gut microbiome and malaria. PLoS Pathog. 2023;19(10):e1011665. doi: 10.1371/journal.ppat.1011665 37824458 PMC10569623

[8] Yooseph S, Kirkness EF, Tran TM, et al. Stool microbiota composition is associated with the prospective risk of *Plasmodium falciparum* infection. BMC Genomics. 2015;16:631. 26296559 10.1186/s12864-015-1819-3 PMC4546150

[9] Huwe T, Prusty BK, Ray A, Lee S, Ravindran B, Michael E. Interactions between parasitic infections and the human gut microbiome in Odisha, India. Am J Trop Med Hyg. 2019;100(6):1486–9. doi: 10.4269/ajtmh.18-0968 30963988 PMC6553895

[10] Easton AV, Raciny-Aleman M, Liu V, Ruan E, Marier C, Heguy A, et al. Immune response and microbiota profiles during coinfection with *Plasmodium vivax* and soil-transmitted helminths. mBio. 2020;11(5):e01705-20. doi: 10.1128/mBio.01705-20 33082257 PMC7587435

[11] Aguilar R, Ubillos I, Vidal M, Balanza N, Crespo N, Jiménez A, et al. Antibody responses to α-Gal in African children vary with age and site and are associated with malaria protection. Sci Rep. 2018;8(1):9999. doi: 10.1038/s41598-018-28325-w 29968771 PMC6030195

[12] Mandal RK, Crane RJ, Berkley JA, Gumbi W, Wambua J, Ngoi JM, et al. Longitudinal analysis of infant stool bacteria communities before and after acute febrile malaria and artemether-lumefantrine treatment. J Infect Dis. 2019;220(4):687–98. doi: 10.1093/infdis/jiy740 30590681 PMC6639600

[13] Mandal RK, Denny JE, Namazzi R, Opoka RO, Datta D, John CC, et al. Dynamic modulation of spleen germinal center reactions by gut bacteria during Plasmodium infection. Cell Rep. 2021;35(6):109094. doi: 10.1016/j.celrep.2021.109094 33979614 PMC8141963

[14] Mukherjee D, Chora ÂF, Mota MM. Microbiota, a third player in the host-plasmodium affair. Trends Parasitol. 2020;36(1):11–8. doi: 10.1016/j.pt.2019.11.001 31787522

[15] Human Microbiome Project Consortium. A framework for human microbiome research. Nature. 2012;486(7402):215–21. doi: 10.1038/nature11209 22699610 PMC3377744

[16] Fontana A, Panebianco C, Picchianti-Diamanti A, Laganà B, Cavalieri D, Potenza A, et al. Gut microbiota profiles differ among individuals depending on their region of origin: an Italian pilot study. Int J Environ Res Public Health. 2019;16(21):4065. doi: 10.3390/ijerph16214065 31652705 PMC6862301

[17] CDC - Malaria - Malaria Worldwide - Impact of Malaria. [cited 2024 Feb 29] Available from: https://www.cdc.gov/malaria/malaria_worldwide/impact.html.

[18] Rudasingwa G, Cho S-I. Determinants of the persistence of malaria in Rwanda. Malar J. 2020;19(1):36. doi: 10.1186/s12936-020-3117-z 31964371 PMC6975052

[19] Taye B, Mekonnen Z, Belanger KD, Davenport ER. Gut-microbiome profiles among soil-transmitted helminths (STHs) infected Ethiopian children enrolled in the school-based mass deworming program. PLoS Negl Trop Dis. 2024;18(10):e0012485. doi: 10.1371/journal.pntd.0012485 39405336 PMC11478818

[20] Loke P, Lim YAL. Helminths and the microbiota: parts of the hygiene hypothesis. Parasite Immunol. 2015;37(6):314–23. doi: 10.1111/pim.12193 25869420 PMC4428757

[21] Lee SC, Tang MS, Lim YAL, Choy SH, Kurtz ZD, Cox LM, et al. Helminth colonization is associated with increased diversity of the gut microbiota. PLoS Negl Trop Dis. 2014;8(5):e2880. doi: 10.1371/journal.pntd.0002880 24851867 PMC4031128

[22] Hodžić A, Dheilly NM, Cabezas-Cruz A, Berry D. The helminth holobiont: a multidimensional host-parasite-microbiota interaction. Trends Parasitol. 2023;39(2):91–100. doi: 10.1016/j.pt.2022.11.012 36503639

[23] Afolabi MO, Ale BM, Dabira ED, Agbla SC, Bustinduy AL, Ndiaye JLA, et al. Malaria and helminth co-infections in children living in endemic countries: a systematic review with meta-analysis. PLoS Negl Trop Dis. 2021;15(2):e0009138. doi: 10.1371/journal.pntd.0009138 33600494 PMC7924789

[24] Degarege A, Veledar E, Degarege D, Erko B, Nacher M, Madhivanan P. *Plasmodium falciparum* and soil-transmitted helminth co-infections among children in sub-Saharan Africa: a systematic review and meta-analysis. Parasit Vectors. 2016;9(1):344. doi: 10.1186/s13071-016-1594-2 27306987 PMC4908807

[25] Schlosser-Brandenburg J, Midha A, Mugo RM, Ndombi EM, Gachara G, Njomo D, et al. Infection with soil-transmitted helminths and their impact on coinfections. Front Parasitol. 2023;2:1197956. doi: 10.3389/fpara.2023.1197956 39816832 PMC11731630

[26] Abbate JL, Ezenwa VO, Guégan J-F, Choisy M, Nacher M, Roche B. Disentangling complex parasite interactions: protection against cerebral malaria by one helminth species is jeopardized by co-infection with another. PLoS Negl Trop Dis. 2018;12(5):e0006483. doi: 10.1371/journal.pntd.0006483 29746467 PMC5963812

[27] Hartgers FC, Yazdanbakhsh M. Co-infection of helminths and malaria: modulation of the immune responses to malaria. Parasite Immunol. 2006;28(10):497–506. doi: 10.1111/j.1365-3024.2006.00901.x 16965285

[28] Global technical strategy for malaria 2016-2030. 2021. [cited 2025 Jan 23] Available from: https://www.who.int/publications/i/item/9789240031357. 10.1186/s12936-016-1302-x PMC4843196 27113588

[29] De Keyzer W, Huybrechts I, De Vriendt V, Vandevijvere S, Slimani N, Van Oyen H, et al. Repeated 24-hour recalls versus dietary records for estimating nutrient intakes in a national food consumption survey. Food Nutr Res. 2011;55:10.3402/fnr.v55i0.7307. doi: 10.3402/fnr.v55i0.7307 22084625 PMC3215303

[30] BMI Z-Score and Percentile Calculator. [cited 2024 Dec 17] Available from: https://www.bcm.edu/bodycomplab/BMIapp/BMI-calculator-kids.html.

[31] Child and Teen BMI Calculator | BMI | CDC. [cited 2024 Dec 17] Available from: https://www.cdc.gov/bmi/child-teen-calculator/index.html.

[32] BMI-for-age (5-19 years). [cited 2024 Dec 17] Available from: https://www.who.int/tools/growth-reference-data-for-5to19-years/indicators/bmi-for-age.

[33] Giemsa Staining of Malaria Blood Films. Malaria Microscopy Standard Operating Procedure-Mm-Sop-07a 1: Purpose And Scope. World Health Organization, 2016. p. 1–6.

[34] Caporaso JG, Lauber CL, Walters WA, Berg-Lyons D, Lozupone CA, Turnbaugh PJ, et al. Global patterns of 16S rRNA diversity at a depth of millions of sequences per sample. Proc Natl Acad Sci U S A. 2011;108 Suppl 1(Suppl 1):4516–22. doi: 10.1073/pnas.1000080107 20534432 PMC3063599

[35] Bolyen E, Rideout JR, Dillon MR, Bokulich NA, Abnet CC, Al-Ghalith GA, et al. Reproducible, interactive, scalable and extensible microbiome data science using QIIME 2. Nat Biotechnol. 2019;37(8):852–7. doi: 10.1038/s41587-019-0209-9 31341288 PMC7015180

[36] Callahan BJ, McMurdie PJ, Rosen MJ, Han AW, Johnson AJA, Holmes SP. DADA2: High-resolution sample inference from illumina amplicon data. Nat Methods. 2016;13(7):581–3. doi: 10.1038/nmeth.3869 27214047 PMC4927377

[37] Edgar RC, Haas BJ, Clemente JC, Quince C, Knight R. UCHIME improves sensitivity and speed of chimera detection. Bioinformatics. 2011;27(16):2194–200. doi: 10.1093/bioinformatics/btr381 21700674 PMC3150044

[38] Rognes T, Flouri T, Nichols B, Quince C, Mahé F. VSEARCH: a versatile open source tool for metagenomics. PeerJ. 2016;4:e2584. doi: 10.7717/peerj.2584 27781170 PMC5075697

[39] Quast C, Pruesse E, Yilmaz P, Gerken J, Schweer T, Yarza P, et al. The SILVA ribosomal RNA gene database project: improved data processing and web-based tools. Nucleic Acids Res. 2013;41(Database issue):D590–6. doi: 10.1093/nar/gks1219 23193283 PMC3531112

[40] Katoh K, Misawa K, Kuma K, Miyata T. MAFFT: a novel method for rapid multiple sequence alignment based on fast Fourier transform. Nucleic Acids Res. 2002;30(14):3059–66. doi: 10.1093/nar/gkf436 12136088 PMC135756

[41] Price MN, Dehal PS, Arkin AP. FastTree 2--approximately maximum-likelihood trees for large alignments. PLoS One. 2010;5(3):e9490. doi: 10.1371/journal.pone.0009490 20224823 PMC2835736

[42] Shannon CE. A mathematical theory of communication. Bell Syst Tech J. 1948;27(3):379–423. doi: 10.1002/j.1538-7305.1948.tb01338.x

[43] Faith DP. Conservation evaluation and phylogenetic diversity. Biol Conserv. 1992;61:1–10.

[44] Chao A. Estimating the population size for capture-recapture data with unequal catchability. Biometrics. 1987;43(4):783–91. doi: 10.2307/2531532 3427163

[45] Pielou EC. The measurement of diversity in different types of biological collections. J Theor Biol. 1966;13:131–144.

[46] Bray JR, Curtis JT. An ordination of the upland forest communities of Southern Wisconsin. Ecol Monogr. 1957;27:325–349.

[47] Lozupone C, Knight R. UniFrac: a new phylogenetic method for comparing microbial communities. Appl Environ Microbiol. 2005;71:8228–35. 16332807 10.1128/AEM.71.12.8228-8235.2005 PMC1317376

[48] Jaccard P. Étude comparative de la distribution florale dans une portion des Alpes et des Jura. Bull Soc Vaudoise Sci Nat. 1901;37:547–79.

[49] Lin H, Peddada SD. Analysis of compositions of microbiomes with bias correction. Nat Commun. 2020;11(1):3514. doi: 10.1038/s41467-020-17041-7 32665548 PMC7360769

[50] R Core Team. R: A Language and Environment for Statistical Computing. R Foundation for Statistical Computing, Vienna, Austria. 2024. Available from: https://www.R-project.org/

[51] Wickham H. ggplot2: Elegant Graphics for Data Analysis. 2016. Available from: doi: 10.1007/978-3-319-24277-4

[52] Hester J, Bryan J. Interpreted String Literals [R package glue version 1.8.0]. CRAN: Contributed Packages. 2024. Available from: doi: 10.32614/CRAN.PACKAGE.GLUE

[53] Wickham H, Averick M, Bryan J, Chang W, McGowan L, François R, et al. Welcome to the Tidyverse. J Open Source Softw. 2019;4(43):1686. doi: 10.21105/joss.01686

[54] Slowikowski K, Schep A, Hughes S, et al. Package “ggrepel” Title Automatically Position Non-Overlapping Text Labels with “ggplot2.” 2024.

[55] Wickham H, François R, Henry L, Müller K, Vaughan D. dplyr: A Grammar of Data Manipulation. CRAN: Contributed Packages. 2014. Available from: doi: 10.32614/cran.package.dplyr

[56] ggExtra - Add marginal histograms to ggplot2, and more ggplot2 enhancements. [cited 27 Jan 2025] Available from: https://cran.r-project.org/web/packages/ggExtra/vignettes/ggExtra.html.

[57] GraphPad Prism version 10.0.0 for Windows, GraphPad Software, Boston, Massachusetts USA. Available from: www.graphpad.com.

[58] Revelle W. psych: Procedures for Psychological, Psychometric, and Personality Research. Northwestern University, Evanston, Illinois. 2023. R package version 2.3.6. Available from: https://CRAN.R-project.org/package=psych.

[59] Wei T, Simko V. R package “corrplot”: Visualization of a Correlation Matrix (Version 0.92). 2021. Available from: https://github.com/taiyun/corrplot.

[60] Hansen MEB, Rubel MA, Bailey AG, Ranciaro A, Thompson SR, Campbell MC, et al. Population structure of human gut bacteria in a diverse cohort from rural Tanzania and Botswana. Genome Biol. 2019;20(1):16. doi: 10.1186/s13059-018-1616-9 30665461 PMC6341659

[61] Tamburini FB, Maghini D, Oduaran OH, Brewster R, Hulley MR, Sahibdeen V, et al. Short- and long-read metagenomics of urban and rural South African gut microbiomes reveal a transitional composition and undescribed taxa. Nat Commun. 2022;13(1):926. doi: 10.1038/s41467-021-27917-x 35194028 PMC8863827

[62] Nguélé AT, Carrara C, Mozzicafreddo M, Chen H, Piersanti A, Salum SS, et al. Association between food or nutrients and gut microbiota in healthy and helminth-infected women of reproductive Age from Zanzibar, Tanzania. Nutrients. 2024;16(9):1266. doi: 10.3390/nu16091266 38732513 PMC11085056

[63] Nyungwe National Park - UNESCO World Heritage Centre. [cited 18 Dec 2024]. Available from: https://whc.unesco.org/en/list/1697/.

[64] Ley RE, Hamady M, Lozupone C, Turnbaugh PJ, Ramey RR, Bircher JS, et al. Evolution of mammals and their gut microbes. Science. 2008;320(5883):1647–51. doi: 10.1126/science.1155725 18497261 PMC2649005

[65] Wiertsema SP, van Bergenhenegouwen J, Garssen J, Knippels LMJ. The Interplay between the gut microbiome and the immune system in the context of infectious diseases throughout life and the role of nutrition in optimizing treatment strategies. Nutrients. 2021;13(3):886. doi: 10.3390/nu13030886 33803407 PMC8001875

[66] Bailén M, Bressa C, Martínez-López S, González-Soltero R, Montalvo Lominchar MG, San Juan C, et al. Microbiota features associated with a high-fat/low-fiber diet in healthy adults. Front Nutr. 2020;7:583608. doi: 10.3389/fnut.2020.583608 33392236 PMC7775391

[67] Gomez-Arango LF, Barrett HL, Wilkinson SA, Callaway LK, Mcintyre HD, Morrison M, et al. Gut microbes low dietary fiber intake increases collinsella abundance in the gut microbiota of overweight and obese pregnant women. Gut Microbes. 2018;9:189–201. 29144833 10.1080/19490976.2017.1406584 PMC6219589

[68] Fu J, Zheng Y, Gao Y, Xu W. Dietary fiber intake and gut microbiota in human health. Microorganisms. 2022;10(12):2507. doi: 10.3390/microorganisms10122507 36557760 PMC9787832

[69] Cantu-Jungles TM, Hamaker BR. Tuning expectations to reality: don’t expect increased gut microbiota diversity with dietary fiber. J Nutr. 2023;153(11):3156–63. doi: 10.1016/j.tjnut.2023.09.001 37690780

[70] De Filippo C, Cavalieri D, Di Paola M, Ramazzotti M, Poullet JB, Massart S, et al. Impact of diet in shaping gut microbiota revealed by a comparative study in children from Europe and rural Africa. Proc Natl Acad Sci U S A. 2010;107:14691–6. 20679230 10.1073/pnas.1005963107 PMC2930426

[71] Rwanda - Comprehensive Food Security and Vulnerability Analysis 2021 - Overview. [cited 24 Mar 2024] Available from: https://microdata.statistics.gov.rw/index.php/catalog/106/study.

[72] Subramanian S, Huq S, Yatsunenko T, Haque R, Mahfuz M, Alam MA, et al. Persistent gut microbiota immaturity in malnourished Bangladeshi children. Nature. 2014;510(7505):417–21. doi: 10.1038/nature13421 24896187 PMC4189846

[73] Afolayan AO, Ayeni FA, Moissl-Eichinger C, Gorkiewicz G, Halwachs B, Högenauer C. Impact of a nomadic pastoral lifestyle on the gut microbiome in the fulani living in Nigeria. Front Microbiol. 2019;10:2138. doi: 10.3389/fmicb.2019.02138 31572342 PMC6753190

[74] Simpson HL, Campbell BJ. Review article: dietary fibre-microbiota interactions. Aliment Pharmacol Ther. 2015;42(2):158–79. doi: 10.1111/apt.13248 26011307 PMC4949558

[75] Van Hul M, Cani PD. The gut microbiota in obesity and weight management: microbes as friends or foe? Nat Rev Endocrinol. 2023;19(5):258–71. doi: 10.1038/s41574-022-00794-0 36650295

[76] Menni C, Jackson MA, Pallister T, Steves CJ, Spector TD, Valdes AM. Gut microbiome diversity and high-fibre intake are related to lower long-term weight gain. Int J Obes. 2017;41:1099–105. 28286339 10.1038/ijo.2017.66 PMC5500185

[77] Palmer C, Bik EM, DiGiulio DB, Relman DA, Brown PO. Development of the human infant intestinal microbiota. PLoS Biol. 2007;5(7):e177. doi: 10.1371/journal.pbio.0050177 17594176 PMC1896187

[78] Bokulich NA, Chung J, Battaglia T, Henderson N, Jay M, Li H, et al. Antibiotics, birth mode, and diet shape microbiome maturation during early life. Sci Transl Med. 2016;8(343):343ra82. doi: 10.1126/scitranslmed.aad7121 27306664 PMC5308924

[79] Vacca M, Celano G, Calabrese FM, Portincasa P, Gobbetti M, De Angelis M. The controversial role of human gut Lachnospiraceae. Microorganisms. 2020;8(4):573. doi: 10.3390/microorganisms8040573 32326636 PMC7232163

[80] Weber AM, Ibrahim H, Baxter BA, Kumar R, Maurya AK, Kumar D, et al. Integrated microbiota and metabolite changes following rice bran intake during murine inflammatory colitis-associated colon cancer and in colorectal cancer survivors. Cancers (Basel). 2023;15(8):2231. doi: 10.3390/cancers15082231 37190160 PMC10136752

[81] Zhou H, Huang D, Sun Z, Chen X. Effects of intestinal Desulfovibrio bacteria on host health and its potential regulatory strategies: a review. Microbiol Res. 2024;284:127725. doi: 10.1016/j.micres.2024.127725 38663233

[82] Lam YY, Ha CWY, Hoffmann JMA, Oscarsson J, Dinudom A, Mather TJ, et al. Effects of dietary fat profile on gut permeability and microbiota and their relationships with metabolic changes in mice. Obesity (Silver Spring). 2015;23(7):1429–39. doi: 10.1002/oby.21122 26053244

[83] Liu C, Wu H, Liu S, Chai S, Meng Q, Zhou Z. Dynamic alterations in yak rumen bacteria community and metabolome characteristics in response to feed type. Front Microbiol. 2019;10:1116. doi: 10.3389/fmicb.2019.01116 31191470 PMC6538947

[84] Kiarie A, Bebora L, Gitao G, Ochien’g L, Okumu N, Mutisya C, et al. Prevalence and risk factors associated with the occurrence of *Campylobacter* sp. in children aged 6-24 months in peri-urban Nairobi, Kenya. Front Public Health. 2023;11:1147180. doi: 10.3389/fpubh.2023.1147180 37808985 PMC10556691

[85] Rennie KL, Coward A, Jebb SA. Estimating under-reporting of energy intake in dietary surveys using an individualised method. Br J Nutr. 97:1169–76. 17433123 10.1017/S0007114507433086

