# Supplementary figures and images for: Gut microbiota composition differences are associated with geographic location and age in malaria-endemic regions of Rwanda
Source: PLoS One. 2025 Jun 3;20(6):e0320698. doi: 10.1371/journal.pone.0320698 (PMC12132938; doi:10.1371/journal.pone.0320698)

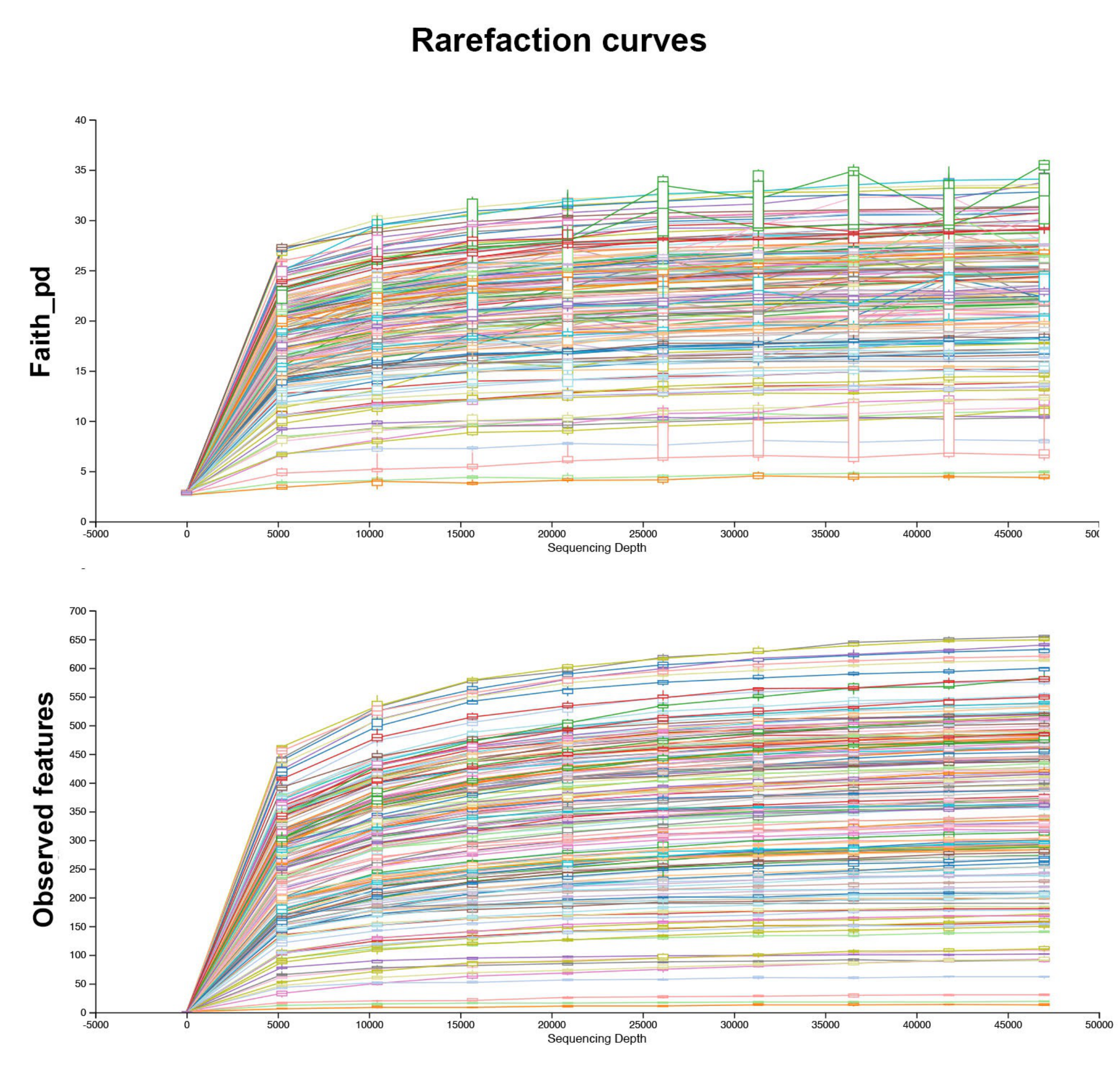

Supplement: S1 Fig — (TIF) [file pone.0320698.s001.tif]

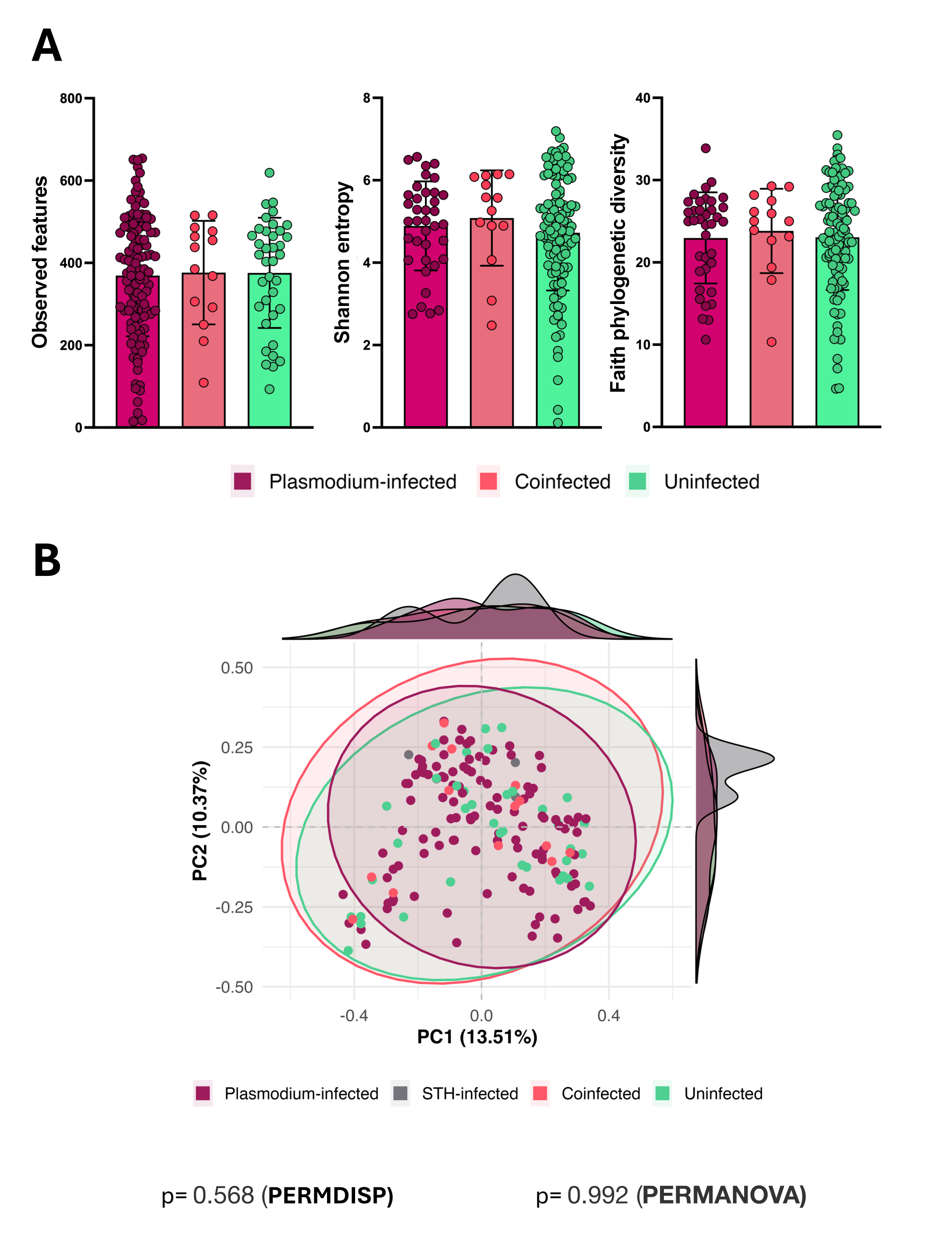

Supplement: S2 Fig — Alpha and beta diversity analyses by infection groups. (TIF) [file pone.0320698.s002.tif]
